# Bypass Patency and Amputation-Free Survival after Popliteal Aneurysm Exclusion Significantly Depends on Patient Age and Medical Complications: A Detailed Dual-Center Analysis of 395 Consecutive Elective and Emergency Procedures

**DOI:** 10.3390/jcm13102817

**Published:** 2024-05-10

**Authors:** Hannah Freytag, Marvin Kapalla, Floris Berg, Hans-Christian Arne Stroth, Tessa Reisenauer, Kerstin Stoklasa, Alexander Zimmermann, Christian Reeps, Christoph Knappich, Steffen Wolk, Albert Busch

**Affiliations:** 1 Department for Vascular and Endovascular Surgery, Klinikum Rechts der Isar, Technical University Munich, 81675 Munich, Germany; 2 Division of Vascular and Endovascular Surgery, Department for Visceral, Thoracic and Vascular Surgery, Medical Faculty Carl Gustav Carus and University Hospital, Technische Universität Dresden, 01307 Dresden, Germany; 3 Department of Vascular Surgery, University Hospital Zürich, 8091 Zürich, Switzerland

**Keywords:** popliteal artery aneurysm, acute limb ischemia, urgent revascularization, major amputation, diameter

## Abstract

**Background/Objectives**: A popliteal artery aneurysm (PAA) is traditionally treated by an open PAA repair (OPAR) with a popliteo–popliteal venous graft interposition. Although excellent outcomes have been reported in elective cases, the results are much worse in cases of emergency presentation or with the necessity of adjunct procedures. This study aimed to identify the risk factors that might decrease amputation-free survival (efficacy endpoint) and lower graft patency (technical endpoint). **Patients and Methods**: A dual-center retrospective analysis was performed from 2000 to 2021 covering all consecutive PAA repairs stratified for elective vs. emergency repair, considering the patient (i.e., age and comorbidities), PAA (i.e., diameter and tibial runoff vessels), and procedural characteristics (i.e., procedure time, material, and bypass configuration). Descriptive, univariate, and multivariate statistics were used. **Results**: In 316 patients (69.8 ± 10.5 years), 395 PAAs (mean diameter 31.9 ± 12.9 mm) were operated, 67 as an emergency procedure (6× rupture; 93.8% severe acute limb ischemia). The majority had OPAR (366 procedures). Emergency patients had worse pre- and postoperative tibial runoff, longer procedure times, and more complex reconstructions harboring a variety of adjunct procedures as well as more medical and surgical complications (all *p* < 0.001). Overall, the in-hospital major amputation rate and mortality rate were 3.6% and 0.8%, respectively. The median follow-up was 49 months. Five-year primary and secondary patency rates were 80% and 94.7%. Patency for venous grafts outperformed alloplastic and composite reconstructions (*p* < 0.001), but prolonged the average procedure time by 51.4 (24.3–78.6) min (*p* < 0.001). Amputation-free survival was significantly better after elective procedures (*p* < 0.001), but only during the early (in-hospital) phase. An increase in patient age and any medical complications were significant negative predictors, regardless of the aneurysm size. **Conclusions**: A popliteo–popliteal vein interposition remains the gold standard for treatment despite a probably longer procedure time for both elective and emergency PAA repairs. To determine the most effective treatment strategies for older and probably frailer patients, factors such as the aneurysm size and the patient’s overall condition should be considered.

## 1. Introduction

A popliteal artery aneurysm (PAA) is the most frequent peripheral artery aneurysm, yet the incidence in the general population is below 1% [1]. Associated risk factors include male gender, age > 55 years, and current smoker. In approx. 60% of patients, both legs are affected [2]. If diagnosed with a PAA, around one-third of patients have at least one additional aneurysm, most frequently in the abdominal (AAA) or thoracic aorta. Vice versa, the prevalence of PAAs ranges from 3 to 11% among AAA patients [3,4].

Compared with AAA, little is known about the specific pathogenesis of PAAs despite the popliteal artery being of a muscular rather than an elastic type [5,6]. However, the clinical presentation and symptoms differ. Rupture is a rare event, yet acute leg ischemia (ALI) due to silent onset long-time or acute peripheral embolisms—or local compression, resulting in deep vein thrombosis or popliteal fossa pain—are more frequent [2,7]. Hence, if not acutely warranting a treatment for symptomatic disease, aneurysm exclusion is advised for a diameter of >20 mm as well as for an eventual severe thrombus load or poor tibial runoff. This approach is currently being investigated to provide better evidence [2,8]. A smaller diameter (<15 mm) upon first diagnosis is associated with more frequent subsequent emergent repairs [8,9]. In general, the annual growth rate varies among individual patients and ranges between 0.7 and 6.5 mm/year, depending on the initial diameter.

Until the 20th century, the aim of treating PAAs was to induce thrombosis by, i.e., proximal and distal ligation, compression (eventually in combination with hip/knee flexion), or specific bandages. The value of revascularization is no longer debated [10]. Depending on the indication (i.e., ALI or elective setting), patient characteristics, and the presence of an adequate saphenous vein, an open PAA repair (OPAR) is considered to be the gold standard of treatment, especially when compared with an endovascular PAA treatment (EPAR) [11]. However, in the emergency setting, additional endovascular procedures such as preoperative lysis and covered stenting have been performed more frequently in recent years [2,11,12]. Additionally, upon good outflow vessels, EPAR can produce equal results, at least in terms of short-term patency [13]. However, comparative or event-randomized studies are missing and questions remain whether the promising short- and long-term results of OPAR, especially in emergent settings, are altered by the patient, aneurysm, or procedural characteristics, paving the way for EPAR [14].

Here, we present a detailed retrospective dual-center 22-year analysis of operative PAA patients, comparing emergent vs. elective OPARs and identifying the negative predictors of amputation-free survival.

## 2. Patients and Methods

### 2.1. Patient Identification and Ethical Approval

All consecutive patients who underwent surgery for PAAs were identified at two university centers from 2000 to 2021 (Munich) and 2005 to 2021 (Dresden), retrospectively based on the electronic patient file systems. Baseline data were retrieved from electronic patient records, follow-up visits, and telephone interviews with family physicians during 2022. For verification, data from previous publications, including approx. 50 patients from one of the centers, were double-checked [15,16].

Patient data were pseudonymized for further analyses. The study was performed in accordance with the Declaration of Helsinki and approved by the local ethics committees (Medical Faculty, Technical University of Munich: 2022-372-S-NP and Technical University Dresden: BO-EK-204042022). The STROBE checklist (v4) for cohort studies was followed as far as possible [17].

### 2.2. Inclusion/Exclusion Criteria

All patients with operations (open/endo) for their PAAs were included. Furthermore, each additional contralateral popliteal artery exceeding 10 mm in diameter was included as a PAA.

Patients with re-do surgery after previous (external/different hospital) attempts to exclude their PAA were excluded. Also, patients with post-traumatic or false aneurysms were excluded. No patients under 18 were diagnosed or included.

### 2.3. Stepwise Analysis and Definitions

The patient baseline characteristics included age, sex, ASA score (American Society of Anesthesiologists: I–V), hypertension, diabetes, smoking status, obesity (body mass index (BMI) > 30), chronic obstructive pulmonary disease (COPD), renal insufficiency (a score ≥ 2 from the Kidney Disease: Improving Global Outcomes (KDIGO) measurement), permanent dialysis, active/previous malignancy, hyperlipidemia, coronary artery disease (CAD), peripheral arterial occlusive disease (PAOD), and known connective tissue disease or vasculitis (no additional diagnostics initiated). Medication was screened for anti-platelets (aspirin/clopidogrel), angiotensin-converting enzyme (ACE) inhibitors, statins, and metformin or insulin administration. The available laboratory parameters included C-reactive protein (CRP), hemoglobin, and the leucocyte count. Data were extracted from the electronic patient records at the time of the index procedure.

Additional aneurysms were identified based on the available medical history of a previous diagnosis and current imaging (CTA, MRI, and ultrasound) and classified as contralateral PAA (>10 mm), AAA (>30 mm), thoracic aortic aneurysm (>40 mm), or iliac/femoral aneurysm (>20 mm). A “dilation phenotype” was defined as multiple aneurysms or a general enlargement of multiple arterial segments to a diameter > 1.5 compared with the native vessel.

The PAA characteristics were the maximum transverse diameter and the number of patent crural outflow vessels before operation as well as asymptomatic and symptomatic disease. The symptoms were defined as a rupture, local pain (no concurrent origin), claudication, tissue loss, deep vein thrombosis (DVT), and an ALI based on the Rutherford classification (I, IIa/b, or III) [11]. All imaging was analyzed by at least two experienced vascular surgeons from the author list. A consensus was reached by an additional review in the case of doubt.

Indication for operation was classified as emergency (TASC IIa or IIb ALI + rupture) vs. elective (including urgent cases operated on within 48 h); an elective indication was based on the diameter (>20 mm), symptoms, or tissue loss related to chronic limb ischemia due to a PAA. Additionally, the time between the operation on the index leg and, eventually, the contralateral limb was assessed.

For operative handling, all surgeries were conducted at the treating surgeon’s discretion; however, the standard procedure was PAA exclusion with a venous bypass interposition. Additional procedures, especially for emergency procedures, were also investigated. Patients with initial stentgraft (EPAR) were also assessed; however, due to the low number, no detailed procedural characteristics were included. Completion angiography after surgery was considered to be the treatment of choice. For ALI, primary fasciotomy was advised. Procedural characteristics included the access (medial/dorsal/conversion), operation time, localization of proximal/distal anastomosis (classified as popliteal–popliteal, distal origin, or femoral artery–popliteal, or crural bypass), material for reconstruction (autologous vein, alloplastic, or composite), number of postoperative patent crural vessels, and the difference between the patent vessels and ankle–brachial index (ABI) before and after surgery. Additional intraoperative procedures were intra-arterial thrombolysis (before and during surgery), embolectomy of target vessels, local endarterectomy preceding the anastomosis, up-/downstream endovascular optimization (balloon angioplasty/stent), immediate revision (i.e., missing pulse, compromised in-/outflow, etc.), additional bypass jump graft, and fasciotomy. Data were extracted from operative charts.

The postoperative course was analyzed for the time in hospital, surgical complications (bleeding, secondary compartment syndrome requiring surgery, surgical-site infection (SSI), bypass occlusion, and nerve lesion), and medical complications (cardiovascular (i.e., myocardial infarction or stroke), pulmonary (i.e., pneumonia or edema), acute kidney failure, and others). Additionally, every surgical procedure on the operated leg during the hospital stay and during follow-up was assessed, including revision for bleeding, SSI, secondary fasciotomy, secondary aneurysm resection, bypass occlusion/stenosis (high grade = bypass at risk), and secondary major amputation.

### 2.4. Endpoints and Outcome Parameters

The primary endpoints were amputation-free survival (efficacy endpoint), comparing emergency vs. elective procedures and primary bypass patency (technical endpoint).

The secondary endpoints were overall survival, reintervention-free survival, secondary patency (after one surgical/interventional revision), and operation time.

Additionally, the timely evolution of the patient, PAA, and procedural characteristics were investigated in three consecutive patient cohorts.

### 2.5. Statistical Analysis

All characteristics with missing information from ≥5 patients are indicated in the respective figure/table and corresponding legends. A statistical analysis was performed using IBM SPSS for Windows, Version 28.0 (IBM Corp., Armonk, NY, USA). All clinical characteristics were grouped to build the categorical or nominal variables. Dichotomous variables were recorded as absolute frequencies (number of cases) and relative frequencies (percentages). Continuous data were presented as the mean ± one standard deviation (SD), unless stated otherwise. Non-symmetrical values were used for the median and interquartile range (IQR). Pearson’s chi-squared or Fisher’s exact test were used to analyze the categorical variables. Differences between means were tested using the *t*-test or Mann–Whitney U test.

Survival and patency data were analyzed using Kaplan–Meier estimates and differences between groups were appointed using the log-rank test. A linear regression was performed to uni-/multivariately analyze the operation time in relation to the dependence of the variables, thus describing the average time difference for each variable. A univariate binary logistic regression analysis was performed to evaluate the influence of the variables on the composite endpoint for amputation-free survival with odds ratios (ORs) and 95% CIs as measures of association. We used a multivariable logistic regression model based on the univariate significant variables. A two-sided *p*-value < 0.05 was considered to be statistically significant in all performed tests.

## 3. Results

### 3.1. Study Cohort, Patient, and PAA Characteristics

**Figure 1:**
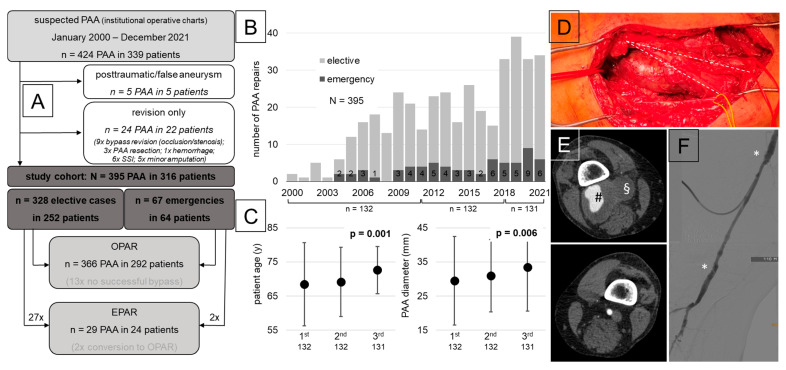
(**A**) Patient selection and final study cohort flow chart. (**B**) Annual increase in emergency and elective procedures and timeline for three consecutive cohorts. (**C**) Distribution of patient age and PAA diameter in three consecutive cohorts (median; interquartile range) (one-way ANOVA; *p* < 0.05 considered to be significant and highlighted in bold) (PAA: popliteal artery aneurysm; SSI: surgical-site infection; OPAR: open PAA repair; EPAR: endovascular PAA repair). (**D**) Intraoperative photograph of 45 mm diameter PAA through the Hunter canal (white dotted line) from the distal femoral artery to segment II of the popliteal artery (red vessel loops) (yellow vessel loops mark the saphenous nerve). (**E**) CT angiography (axial view) with giant partially thrombosed PAA (**#**) on the right leg (**upper picture**) with dilation of the popliteal vein (**§**) due to hindered outflow in comparison with a normal configuration on the contralateral leg (**lower picture**). (**F**) Intraoperative fluoroscopy after popliteo–popliteal venous graft interposition (asterisk: proximal/distal anastomosis) via puncture through the upper anastomosis with consecutive two-vessel tibial runoff (fibular + anterior tibial artery).

Overall, 395 PAAs were operated on in 316 patients during the 22-year study period (Figure 1A). Of these, 195 patients had bilateral disease upon the first diagnosis and 79 patients had both legs operated on (Figure 1A; Table S1). Based on the index leg procedure, 64 patients presented as an emergency and 3 patients had consecutive emergency procedures on both legs (Table 1 and Table S1). Emergency patients were significantly older and had a worse preoperative tibial runoff. However, the vessel diameter was not different from elective cases (Table 1 and Table S1). A PAA rupture was very rare (six cases) and a severe ALI was the most frequent cause for an emergency operation (93.8%). Half of the elective patients had symptoms of claudication (26.6%) or popliteal fossa pain (19.4%).

The annual number of treated PAAs increased over the study period from 2 procedures in 2000 to 34 in 2021 (Figure 1B). In the three consecutive periods, the patient age significantly increased from 68.1 ± 10.4 to 72.5 ± 9.7 years (*p* = 0.001) and the average maximum diameter increased from 29.5 ± 13 to 33.4 ± 12.8 mm (*p* = 0.006). However, both parameters were significantly correlated (R = 0.21; *p* = 0.001) (Figure 1C and Figure S1A; Table S1). Otherwise, the patient and PAA characteristics or emergency presentation did not show significant changes over time (Figure 1B; Table S1).

### 3.2. Procedural Analysis

A primary EPAR was performed to treat 29 PAAs in a minority of 24 patients (7.6%), twice in an emergency setting (Figure 1A). The indications were, i.e., a poor cardiac function or specific surgeons’ or patients’ preferences at the time. The PAA and procedural subgroup data are shown in Table S2.

Completion angiography was available for over 80% of all patients. When further investigating the 366 OPAR procedures, only 13 (3.6%) were not successful, mostly during emergency revascularization (Figure 1A; Table 2). Generally, emergency procedures had a significantly longer procedure time (+42.6 min), exclusively medial access (98.4%), a higher number of longer reconstructive bypasses (45.3%) with a higher percentage of alloplastic material (35.4%) used, and more additional procedures such as intra-arterial lysis, embolectomy, and simultaneous fasciotomy (Table 2). Despite a slight increase in the mean procedure time from 225 (209–247) to 259 (242–287) min (*p* = 0.002) and a non-significant trend towards more emergency procedures, these characteristics did not change over time (Figure 1B; Tables S1 and S3).

**Figure 2:**
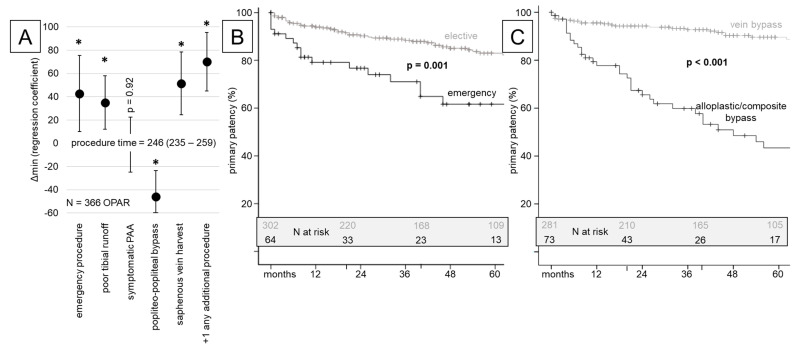
(**A**) Multivariate regression analysis of procedure time for OPAR procedures. The median procedure time was 246 (235–259) min. The regression coefficient displays the prolongation or reduction in the operation (values are given in Table S4; *: *p* < 0.05). (**B**,**C**) Kaplan–Meier estimate plots for the technical endpoint primary patency over 60 months stratified for elective vs. emergency revascularization (**B**) and vein vs. other material bypasses (**C**), respectively. Patient numbers at risk are displayed as an inlet (*p* < 0.05 considered to be significant and highlighted in bold).

A multivariate regression analysis revealed that despite an emergency setup, a poor tibial runoff (+34.9 min) and the necessity of additional procedures (+70.1 min) significantly prolonged the total procedure time (Figure 2A; Table S4). The most frequently used popliteal–popliteal bypass configuration was the fastest (−45.9 min) and the harvesting of the saphenous vein significantly prolonged the procedure time by 51.4 min (Figure 2A).

### 3.3. Operative Results

The hemodynamic data (tibial vessel runoff difference and ABI) were available for 303 procedures (82.8%). Here, more vessels could be recruited in emergency patients (0.64 ± 0.93 vs. 0.19 ± 0.95; *p* < 0.001), but the average total number was still higher in elective procedures, both pre- and postoperatively (Table 1 and Table 2). Emergency patients had a significantly longer hospital stay (median 7 vs. 17.5 days; *p* < 0.001) and approx. 5× more medical and 2× more surgical complications, specifically reoperations due to bypass occlusion/high-grade stenosis, major amputations, and wound revisions (Table 2). Three patients died during the hospital stay (2× myocardial infarction, 1× sepsis) (Table 3).

### 3.4. Long-Term Endpoint Analysis

The median follow-up was 49 months (range 46–56), with only twelve patients lost to follow-up. No differences regarding the number of reoperations were seen for emergency patients (Table 2). After 14 major amputations during the initial stay, 7 more were registered during follow-up. The primary (86.2% vs. 70.9%; *p* < 0.001) and secondary (96.6% vs. 87.5%; *p* = 0.003) patency rates for OPAR after five years were high, yet significantly better for elective procedures (Table 3; Figure 2B and Figure S1B). Again, these results did not change over consecutive cohorts (Table S5).

Regarding the bypass material, the saphenous vein was used most frequently and both graft patency and reintervention-free survival (technical endpoint) were significantly better (each *p* < 0.001) compared with composite and alloplastic grafts (Figure 2C and Figure S1C).

**Figure 3:**
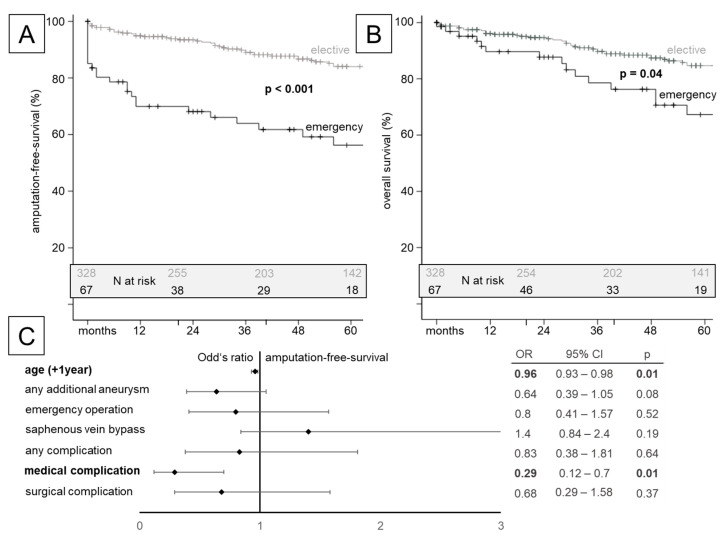
Kaplan–Meier estimate plots for the efficacy endpoint of amputation-free survival (**A**) and overall survival (**B**) stratified for elective vs. emergency repair, respectively. Patient numbers at risk are displayed as an inlet. (**C**) Multivariate risk analysis to reach the efficacy endpoint for amputation-free survival (OR: odds ratio; CI: 95% confidence interval; *p* < 0.05 considered to be significant and highlighted in bold; values are given in Table S6).

Overall, 90 patients died; however, the numbers at risk during the longer-term follow-up rapidly decreased (Table 3). For both the amputation-free (efficacy endpoint) and overall survival, a significantly better outcome was seen for elective procedures (Figure 3A,B). The multivariate analysis revealed that the odds ratio to reach this efficacy endpoint was significantly reduced if the patients were older or suffered from a medical complication during their initial hospitalization (Figure 3C; Table S6).

## 4. Discussion

This study conclusively demonstrates that a popliteo–popliteal saphenous vein graft as the treatment of choice has the best short- and long-term results regarding bypass patency and amputation-free survival in PAA patients. Patient, procedural, and outcome characteristics have only minimally changed over the last years, with more operations being performed on older patients with possibly slightly bigger aneurysms. Unique to this study, a detailed procedural time analysis revealed the effects of, i.e., an emergency setting or additional intraoperative measures.

Patients becoming older and possibly frailer has been a trend for many years in different vascular procedures, especially due to an aging society and the incorporation and availability of more endovascular procedures [13,18,19]. For aortic procedures, an increasing age has been repeatedly demonstrated to be a negative predictor, regardless of the technique used [20]. For open surgery in chronic and acute limb ischemia, controversial data have been reported for both peripheral arterial disease and PAA. Generally, open or endovascular revascularization is safe, including for older patients; in the setting of ALI, an increase in the patient age is a major negative predictor for short- and mid-term limb and patient survival, as demonstrated here as well as by others [14,16,19,21]. Concordantly, medical complications during the postoperative hospital stay seem to have a major influence on these outcomes. Johnson et al. additionally identified intraoperative red blood cell transfusion as a negative predictor in their large-scale analysis [22]. Some groups reported additional aneurysmorrhaphy as part of their standard treatment; this was not performed on our patients [23]. Similar to other studies of this kind, an elective repair using vein grafts has demonstrated the best results with low early and late reintervention and amputation rates [12,13,19,22,24].

Patients operated on as an emergency, mostly due to severe ALI, had the worst outcome in this study and others [12,14]. Generally, a poor vessel runoff is responsible. Although additional procedures such as thrombolysis to improve outflow have been reported, the results are controversial and the complication rates seem to be considerate [11,25,26]. Thus, the question remains of how to avoid such emergency treatments. Although routine screening is recommended in the susceptible population (i.e., men with AAA), general screening will never be applicable [27]. The evaluation of tibial vessel runoff (≤2) and PAA contortion (>45°) have been suggested as alternative cut-off values to indicate surgical repair [12,22]. However, the number of patients included in these studies were low. Interestingly, data on the association between intraluminal thrombus and patent outflow vessels are scarce, despite an obvious connection [28]. Future studies taking into account the aneurysm volume, i.e., thrombus/lumen ratio, could help to broaden the indicatory spectrum in an analogy to AAA, where the aneurysm volume, regardless of ILT, has been suggested to be a more reliable indicator of future repair [9,29].

Based on the data reported, a primary EPAR, despite being increasingly used, might not be justified as a general approach, specifically due to lower patency rates and the need for re-do procedures [13,14,19,23]. This might be due to an unfavorable mural thrombus apposition in the stented zones, especially with longer coverage, even with stentgrafts with high flexibility [24,30,31]. However, the value of EPARs might need to be reconsidered in the setting where no venous graft is available. Given the clearly demonstrated inferiority of such reconstructions, a Viaban^©^ prosthesis might be more suitable [32]. A new technical approach has been reported that adapts a flow diversion applied to intracranial aneurysms using bare metal nitinol stents with or without additional coils deployed in the aneurysm sac; this warrants further research [33].

Our study was naturally limited by its retrospective nature and the low number of EPAR procedures compared with open procedures as well as undetermined indications for primary stentgrafts. Additionally, patients were lost to follow-up, especially regarding the mid- and long-term ranges. Hence, no progress on the superiority/inferiority of one treatment over another can be provided. Similarly, due to the low number of major amputations, no further statistical analysis could be performed. The same accounts for the even lower number of female patients. Recent registry data suggest that women might be more severely affected at a lower PAA diameter, again in accordance with AAA patients [34,35].

Although the number of female PAA patients cannot be altered, a future study design could account for this. A blinded 1:1 randomization to OPAR vs. EPAR procedures is desirable and, based on all the retrospective cohort and registry data provided, should probably focus on a selected patient group [9,13,19,22,23]. Here, the value of EPAR techniques on advanced ages and emergency cases should be evaluated. Such a specific study design could help to reduce bias and establish causality.

## 5. Conclusions

Open surgery for PAA is a classic approach with excellent short- and long-term results for this rare clinical problem, especially if treated with a saphenous vein graft interposition. Emergency procedures are challenging and are complicated by the need for a greater technical armamentarium. However, amputation-free survival is most significantly influenced by patient age and in-hospital medical complications. Thus, for older and frailer patients, endovascular means might offer a survival benefit of yet unclear significance.

## Figures and Tables

**Table 1:** Patient and PAA characteristics at first operation (index leg).

			Combined n = 316	Elective n = 252	Emergency n = 64	*p*-Value
**Patient Characteristics**						
	Age (years; mean ± SD)		69.8 ± 10.5	69 ± 10.2	72.8 ± 11.2	**0.009**
	Sex (male; N, %)		305 (96.5)	243 (96.4)	62 (96.9)	0.86
	ASA score	I and II	114 (36.1)	98 (38.9)	16 (25)	0.055
		III	197 (62.3)	152 (60.3)	45 (70.3)	
		IV and V	3 (0.9)	1 (0.4)	2 (3.1)	
Comorbidities	Obesity (BMI > 30)		62 (23.2)	52 (24.3)	10 (18.9)	0.4
	Hypertension		257 (81.6)	204 (81.3)	53 (82.8)	0.78
	Diabetes		72 (22.9)	52 (20.7)	20 (31.3)	0.07
	Hyperlipidemia		202 (64.1)	166 (66.1)	36 (56.3)	0.14
	CAD		121 (38.4)	91 (36.3)	30 (46.9)	0.12
	Renal insufficiency		66 (21.8)	44 (18.3)	22 (34.9)	**0.005**
	Dialysis		1 (0.3)	-	1 (1.6)	0.51
	COPD		17 (5.4)	12 (4.8)	5 (8.1)	0.31
	PAOD		122 (39)	105 (42)	17 (27)	**0.03**
	Malignancy		45 (14.8)	32 (13.1)	13 (21.3)	0.1
	Nicotine abuse (current)		79 (26.2)	61 (25.2)	18 (28.6)	0.45
	Nicotine abuse (ex) *^A^		121 (47.3)	100 (39.1)	21 (32.8)	0.49
Aneurysm	AAA		109 (34.9)	91 (36.5)	18 (28.6)	0.24
	TAA		20 (7.1)	16 (6.9)	4 (7.8)	0.81
	Iliac/femoral artery		89 (29)	73 (29.7)	16 (26.2)	0.59
	Dilation phenotype		55 (17.9)	46 (18.6)	9 (15)	0.51
Medication	ASS/clopidogrel		201 (64.4)	162 (65.1)	39 (61.9)	0.64
	ACE inhibitor		131 (42)	110 (44.2)	21 (32.8)	0.12
	Statins		149 (47.8)	119 (47.8)	30 (46.9)	0.98
	Metformin		28 (9)	23 (9.2)	5 (7.9)	0.75
	Insulin		11 (3.5)	7 (2.8)	4 (6.3)	0.17
Serum	CRP (mg/dL; mean ± SD)		1.6 ± 2.9	1.1 ± 2	3.1 ± 4.7	**<0.001**
	Hb (g/dL; mean ± SD)		14 ± 1.9	14.1 ± 1.8	13.7 ± 2.1	0.21
	Leucocytes (G/L; mean ± SD)		8.1 ± 3.4	7.6 ± 2.4	9.9 ± 5.5	**<0.001**
**PAA Characteristics**						
	Diameter (mm; mean ± SD)		31.9 ± 12.9	31.7 ± 12.6	32.6 ± 14.7	0.91
	Bilateral disease		195 (61.7)	150 (59.5)	45 (70.3)	0.11
	Operation contralateral limb		79 (25)	76 (30.1)	3 (4.7)	**<0.001**
	Diameter contralateral PAA		29.2 ± 8.9	29.1 ± 8.5	30.7 ± 8.9	0.7
	Time between limbs (months)		5 (4–12)	5 (4–13)	4 (3–15)	**0.05**
	Tibial runoff vessels *^B^		0:42 (16.7)	0:21 (10.3)	0:21 (43.8)	**<0.001**
			1:59 (23.4)	1:47 (23)	1:12 (25)	
			2:61 (24.2)	2:54 (26.5)	2:7 (14.6)	
			3:90 (35.7)	3:82 (40.2)	3:8 (16.7)	
	Symptomatic		211 (66.8)	147 (58.3)	64 (100)	**<0.001**
Symptoms	Rupture		6 (1.9)	-	6 (9.4)	**-**
	Local pain		57 (18)	49 (19.4)	8 (12.5)	0.20
	Claudication		67 (21.2)	67 (26.6)	-	**<0.001**
	Tissue loss		8 (2.5)	6 (2.4)	2 (3.1)	0.745
	DVT		2 (0.6)	2 (0.8)	-	0.48
	Ischemia	TASC I	39 (39.4)	39 (15.5)	-	**<0.001**
		TASC IIa	24 (24.2)	-	24 (37.5)	
		TASC IIb	34 (34.3)	-	34 (53.1)	
		TASC III	2 (2)	-	2 (3.3)	

Values presented as absolute numbers and percentage or mean ± one standard deviation. ASA: American Society of Anesthesiology; BMI: body mass index; CAD: coronary heart disease; renal insufficiency = serum creatinine > 1.2 mg/dL; COPD: chronic obstructive pulmonary disease; PAOD: peripheral arterial occlusive disease; AAA: abdominal aortic aneurysm; TAA: thoracic aortic aneurysm; ASS: aspirin; ACE: angiotensin-converting enzyme; CRP: C-reactive protein; Hb: hemoglobin; DVT: deep vein thrombosis; TASC: Transatlantic Society Consensus classification of acute limb ischemia (patients may have presented with ≥1 symptoms). Chi-squared and Mann–Whitney tests used to compare elective vs. emergency cohort; *p* < 0.05 considered to be significant and highlighted in bold; * calculation based on numbers given (*A: 256 patients, 81.0%; *B: 252 patients, 79.8%).

**Table 2:** Procedural details and postoperative surgical course.

			Combined n = 366	Elective n = 302	Emergency n = 64	*p*-Value
**Surgical Details**						
	Procedure time (min, median)		246 (235–259)	242 (231–254)	298 (278–325)	**0.002**
Bypass configuration	Medial access (vs. dorsal)		316 (86.3)	256 (84.8)	63 (98.4)	**0.003**
	Popliteo–popliteal		223 (60.9)	197 (65.2)	26 (40.6)	**0.023**
	Distal origin–popliteal		66 (18.0)	53 (17.5)	13 (20.3)	
	Crural bypass		65 (17.8)	49 (16.2)	16 (25)	
	No successful bypass		13 (3.6)	3 (1)	10 (15.6)	**<0.001**
	Material	Saphenous vein	281 (76.8)	249 (82.5)	32 (50)	**<0.001**
		Alloplastic	64 (18)	47 (15.5)	17 (26.2)	
		Composite	9 (2.5)	3 (1)	6 (9.2)	
Add-on procedures	Lysis pre-/intraop		28 (10.4)	13 (4.3)	15 (23.4)	**<0.001**
	Embolectomy		76 (20.7)	34 (11.2)	42 (64.6)	**<0.001**
	Local TEA		31 (8.4)	27 (8.9)	4 (6.2)	0.47
	PTA/stent (up-/downstream)		37 (10.1)	29 (9.6)	8 (12.3)	0.51
	Immediate revision		57 (15.5)	45 (14.9)	12 (18.5)	0.47
	Additional (jump) graft		9 (2.4)	7 (2.3)	2 (3.1)	0.72
	Fasciotomy		32 (8.7)	5 (1.7)	27 (41.5)	**<0.001**
**Hemodynamic Changes**						
	Runoff vessels, postoperative *		0:15 (5)	0:5 (2)	0:10 (18.2)	**<0.001**
			1:88 (28.1)	1:69 (26.6)	1:19 (34.5)	
			2:87 (28.7)	2:73 (29.4)	2:14 (25.5)	
			3:113 (37.3)	3:101 (40.7)	3:12 (21.8)	
	Δ Runoff (pre/post; mean ± SD)		0.27 ± 0.96	0.19 ± 0.95	0.64 ± 0.93	**<0.001**
	Δ ABI (pre/post; mean ± SD)		0.18 ± 0.35	0.12 ± 0.29	0.6 ± 0.42	**<0.001**
**Postoperative Course** (**In-Hospital**)						
	Hospital stay (d; median)		8 (8–10)	7 (7–8)	17.5 (12–23)	**<0.001**
	Surgical complication		104 (28.5)	75 (24.8)	29 (45.3)	**<0.001**
	Bleeding		12 (3.3)	8 (2.6)	4 (6.2)	
	Compartment syndrome		8 (2.2)	4 (1.3)	4 (6.2)	
	Nerve lesion		5 (1.4)	1 (0.3)	4 (6.2)	
	SSI		63 (17.4)	43 (14.2)	20 (31.3)	
	Medical complication		42 (11.5)	20 (6.6)	22 (34.4)	**<0.001**
	Cardiovascular		15 (4.1)	7 (2.3)	8 (12.3)	
	Pulmonary		8 (2.2)	1 (0.3)	7 (10.8)	
	Acute kidney failure		10 (2.7)	4 (1.3)	6 (9.4)	
	Other		24 (6.5)	14 (4.6)	10 (15.4)	
Reoperation	Hemorrhage		12 (3.3)	8 (2.6)	4 (6.2)	0.15
	Compartment syndrome		7 (1.9)	4 (1.3)	3 (4.6)	0.078
	Bypass occlusion		9 (2.5)	4 (1.3)	5 (7.8)	**0.002**
	Bypass stenosis		7 (1.9)	3 (1)	4 (6.2)	**0.004**
	Aneurysm resection		3 (0.8)	2 (0.7)	1 (1.5)	0.48
	Amputation (major)		14 (3.8)	2 (0.7)	12 (18.5)	**<0.001**
	Wound revision		43 (11.8)	24 (7.9)	19 (29.7)	**<0.001**
**Follow-Up**						
	Follow-up (months, median IQR)		49 (46–56)	51.5 (46–58)	40 (28–53)	**0.034**
**Postdischarge Course** (**Follow-Up**)						
Reoperation	Hemorrhage		6 (1.6)	5 (1.7)	1 (1.5)	0.94
	Bypass occlusion/stenosis		98 (26.8)	79 (26.2)	19 (29.7)	0.56
	Aneurysm resection		17 (4.6)	17 (5.6)	-	0.070
	Amputation		6 (1.7)	4 (1.3)	2 (3.1)	0.22
	Wound revision		34 (9.2)	25 (8.3)	9 (13.8)	0.063

Values presented as absolute numbers and percentage, mean ± one standard deviation, or median with interquartile range (IQR); three patients had conversion from dorsal to medial access (shown with medial). TEA: thrombendarterectomy; PTA: percutaneous transluminal angioplasty; ABI: ankle brachial index. Chi-squared and Mann–Whitney tests used to compare elective vs. emergency cohort; *p* < 0.05 considered to be significant and highlighted in bold; * calculation based on numbers given (*: 303 procedures, 82.8%).

**Table 3:** Outcome analysis.

		Study Cohort N = 395	EPAR	OPAR			
			n = 29	Combined n = 366	Elective n = 302	Emergency n = 64	*p*-Value
**Major Amputation**							
In-hospital		14 (3.6)	-	14 (3.8)	2 (0.7)	12 (18.5)	**<0.001**
1 year		18 (4.6)	-	18 (4.9)	4 (1.3)	14 (21.8)	**<0.001**
5 years		18 (4.6)	-	18 (4.9)	4 (1.3)	14 (21.8)	
Overall		21 (5.3)	1 (3.4)	20 (5.5)	6 (2)	14 (22.6)	**<0.001**
**Mortality**							
In-hospital		3 (0.8)	-	3 (0.8)	1 (0.3)	2 (3.1)	**0.008**
1 year		16 (4.1)	1 (3.4)	15 (4.1)	11 (3.6)	4 (6.3)	0.072
5 years		50 (12.6)	3 (10.3)	47 (12.8)	34 (11.3)	13 (20.3)	
Overall		90 (22.8)	8 (27.6)	82 (22.5)	64 (21.3)	18 (28.1)	0.254
**Patency**							
Primary	In-hospital	370 (93.7)	29 (100)	343 (97.5)	294 (97.3)	49 (90.7)	**0.002**
	1 year	348 (88.1)	24 (82.8)	325 (92.1)	282 (93.3)	43 (79.6)	**<0.001**
	5 years	316 (80)	19 (65.5)	297 (84.1)	258 (86.2)	39 (70.9)	**<0.001**
	Overall	299 (75.7)	15 (51.7)	284 (80.2)	246 (82.3)	38 (69.1)	**0.016**
Sec	1 year	379 (95.9)	29 (100)	352 (96.2)	296 (98.1)	56 (87.5)	**0.003**
	5 years	374 (94.7)	26 (89.7)	348 (95.1)	292 (96.6)	56 (87.5)	**0.01**
	Overall	358 (90.6)	25 (86.2)	333 (93.3)	284 (94.7)	49 (86)	**0.016**

EPAR: endovascular PAA repair; OPAR: open PAA repair; sec: secondary; *p* < 0.05 considered to be significant and highlighted in bold.

## Data Availability

All research data for this study are included in the manuscript or the Supplementary Materials.

## Supplementary Materials

The following supporting information can be downloaded at: https://www.mdpi.com/article/10.3390/jcm13102817/s1, Figure S1: (A) Pearson correlation for PAA diameter and patient age at surgery (*p* < 0.05 is considered significant and highlighted bold). (B) Kaplan-Meier plot for secondary patency over 60 months stratified for elective vs. emergency revascularization. Patient numbers at risk are displayed as inlet. (C) Kaplan-Meier plot for re-intervention free survival over 60 months stratified for vein vs. other material bypass revascularization; Table S1: Patient and PAA characteristics by consecutive thirds. Values presented as absolute numbers and percentage or mean ± one standard deviation; ASA = American society of anesthesiology, BMI = body mass index, CAD = coronary heart disease, renal insufficiency = serum creatinine > 1.2 mg/dL COPD = chronic obstructive pulmonary disease; PAOD = peripheral arterial occlusive disease; AAA = Abdominal Aortic Aneurysm; TAA = thoracic aortic aneurysm; ASS = aspirin; ACE = angiotensin converting enzyme; CRP = C reactive protein, Hb = hemoglobin; DVT = deep vein thrombosis; TASC = Transatlantic Society Consensus classificiation of acute limb ischemia; chi square or 1-way anova test to compare cohorts, *p* < 0.05 is considered significant and highlighted bold; * calculation based on numbers given (*: 303 procedures: 82.8%); Table S2: Primary EPAR indication, patient, PAA characteristics and procedural details. Values presented as absolute numbers and percentage or mean ± one standard deviation; BMI= body mass index, CAD = coronary heart disease, renal insufficiency = serum creatinine > 1.2 mg/dL, COPD = chronic obstructive pulmonary disease; PAOD = peripheral arterial occlusive disease; DVT = deep vein thrombosis; TASC = Transatlantic Society Consensus classificiation of acute limb ischemia; EPAR = endovascular PAA repair, OPAR = open PAA repair; * calculation based on numbers given (*: 24 procedures: 83.7%); Table S3: Procedural details OPAR by consecutive thirds. Values presented as absolute numbers and percentage, mean ± one standard deviation or median with interquartile range [IQR]; three patients had conversion from dorsal to medial access (shown with medial); TEA = thrombendarterectomy, PTA = percutaneous transluminal angioplasty, ABI = ankle brachial index; chi square or 1-way ANOVA test to compare cohorts, *p* < 0.05 is considered significant and highlighted bold;. * calculation based on numbers given (*: 303 procedures: 82.8%); Table S4: Univariate and multivariate operating time analysis of all 366 OPAR procedures. x = Inclusion in the multivariate model; *p* < 0.05 is considered significant and highlighted bold; Table S5: OPAR outcome by consecutive cohorts. EPAR = endovascular PAA repair, OPAR = open PAA repair, sec = secondary; *p* < 0.05 is considered significant and highlighted bold; Table S6: Univariate and multivariate outcome analysis on primary endpoint “amputation-free survival” of entire PAA cohort. ASA = American society of anesthesiology; EPAR = endovascular PAA repair, OPAR = open PAA repair; x = inclusion in the multivariate model; *p* < 0.05 is considered significant and highlighted bold.
