# Supplementary material for: Bypass Patency and Amputation-Free Survival after Popliteal Aneurysm Exclusion Significantly Depends on Patient Age and Medical Complications: A Detailed Dual-Center Analysis of 395 Consecutive Elective and Emergency Procedures
Source: J Clin Med. 2024 May 10;13(10):2817. doi: 10.3390/jcm13102817 (PMC11122537; doi:10.3390/jcm13102817)
Supplement: Supplementary file 1 [file jcm-13-02817-s001.zip › jcm-2954549-supplementary.pdf]

Supplement Material

Supplement Figure and Supplement Figure Legend

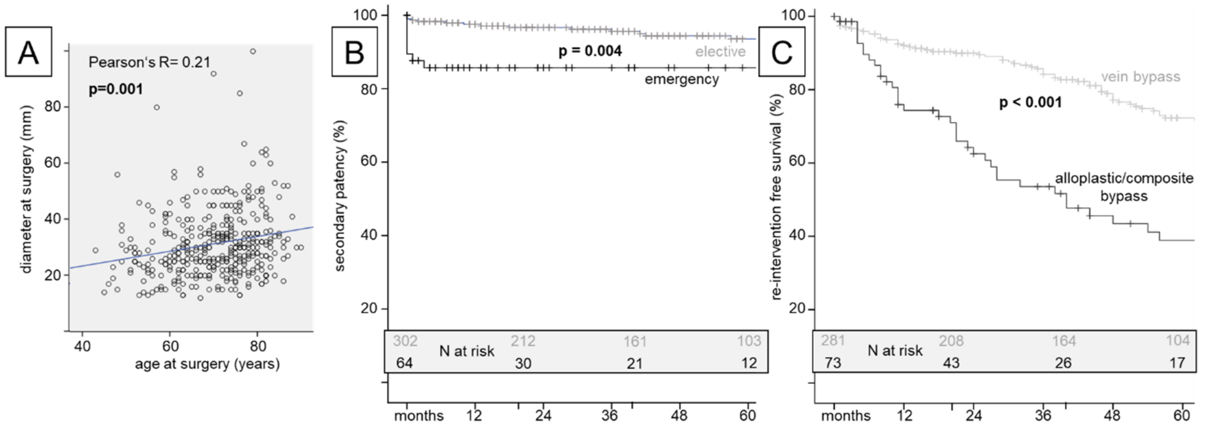

Figure S1. (A) Pearson correlation for PAA diameter and patient age at surgery ($p < 0.05$ is considered significant and highlighted bold). **(B)** Kaplan-Meier plot for secondary patency over 60 months stratified for elective vs. emergency revascularization. Patient numbers at risk are displayed as inlet. **(C)** Kaplan-Meier plot for re-intervention free survival over 60 months stratified for vein vs. other material bypass revascularization.

Supplement Tables

			**combined**<br>n=395	1st third<br>n=132	2nd third<br>n=132	3rd third<br>n=131	trend	p
**patient characteristics**								
	age (years; mean ± SD)		72 ± 10.2	68.4 ± 12.2	69.1 ± 10.2	72.6 ± 6.9	↑	**0.001**
	sex (male: N; %)		382 (96.7)	127 (96.2)	127 (96.2)	128 (97.7)	↑	0.74
	ASA score (I- VI median)		III	III	III	III	=	0.55
comorbidities	obesity (BMI >30)		75 (22.1)	20 (25)	26 (20)	29 (22.5)	=	0.7
	hypertension		326 (82.7)	113 (86.3)	111 (84.1)	102 (77.9)	↓	0.186
	diabetes		86 (21.8)	26 (19.8)	32 (24.2)	28 (21.4)	=	0.68
	hyperlipidemia		253 (64.2)	85 (64.9)	86 (65.2)	82 (62.6)	=	0.89
	CAD		155 (39.3)	48 (36.6)	58 (43.9)	49 (37.4)	=	0.41
	renal insufficiency		76 (19.9)	25 (20.7)	20 (15.3)	31 (24)	=	0.21
	dialysis		1 (0.3)	1 (0.8)	-	-		0.32
	COPD		21 (5.4)	12 (9.2)	5 (3.8)	4 (3.1)	↓	0.069
	PAOD		147 (37.5)	50 (38.2)	49 (37.1)	48 (37.2)	=	0.98
	malignancy		58 (15.1)	19 (14.5)	23 (17.4)	16 (13.2)	=	0.63
	nicotine abuse (active)		102 (26.9)	36 (29.5)	38 (29)	28 (22.2)	↓	0.35
	nicotine abuse (ex)		150 (47.5)	60 (52.2)	51 (50.5)	39 (39)	↓	0.12
aneurysm	AAA		139 (35.6)	49 (38.3)	41 (31.1)	49 (37.7)	=	0.40
	TAA		24 (6.7)	9 (7.7)	4 (3.2)	11 (9.5)	=	0.13
	iliac/femoral artery		111 (28.9)	47 (37.6)	32 (24.8)	32 (24.6)	↓	**0.033**
	dilation phenotype		79 (20.6)	38 (29.9)	33 (25.6)	8 (6.3)	↓	**<0.001**
medication	ASS/Clopidogrel		250 (64.1)	85 (66.4)	80 (60.6)	85 (65.4)	=	0.58
	ACE inhibitor		173 (44.4)	57 (44.5)	67 (50.8)	49 (37.7)	=	0.10
	Statins		184 (47.2)	46 (35.9)	70 (53)	68 (52.3)	↑	**0.008**
	Metformin		33 (8.5)	12 (9.4)	13 (9.8)	8 (6.2)	=	0.51
	Insulin		14 (3.6)	1 (0.8)	8 (6.1)	5 (3.8)	=	0.072
serum	CRP (mg/dL mean ± SD)		1.5 ± 2.8	1.5 ± 2.9	1.5 ± 2.5	1.6 ± 3.1	=	0.52
	Hb (g/dl, mean ± SD)		14.3 ± 6.6	15 ± 11.4	13.8 ± 1.7	14.1 ± 2.3	=	0.75
	leucocytes (G/L, mean ± SD)		8.1 ± 4.7	8.1 ± 6.4	8.2 ± 4.3	7.9 ± 2.9	=	0.95
**PAA characteristics**								
	diameter (mm; mean ± SD)		31.9 ± 12.9	29.5 ± 13	30.9 ± 10.6	33.4 ± 12.8	↑	**0.006**
	bilateral disease		276 (69.9)	94 (71.2)	93 (70.5)	89 (67.9)	=	0.83
	emergency presentation		67 (17.0)	19 (14.4)	23 (17.4)	25 (19.1)	↑	0.59
	tibial runoff vessels * (#)		0: 59 (60)<br>1: 73 (23.2)<br>2: 74 (23.6)<br>3: 116 (37.1)	0: 17 (17.5)<br>1: 25 (25.8)<br>2: 24 (24.7)<br>3: 31 (32)	0: 11 (11.3)<br>1: 20 (20.6)<br>2: 19 (19.6)<br>3: 47 (48.5)	0: 22 (18.5)<br>1: 28 (23.5)<br>2: 31 (26.1)<br>3: 38 (31.9)		0.21
	symptomatic		229 (57.9)	78 (59.1)	75 (56.8)	76 (58)	=	0.38
symptoms	rupture		6 (1.5)	2 (1.5)	-	4 (3.1)		0.61
	local pain		58 (14.7)	17 (12.9)	16 (12.1)	25 (19.1)	↑	0.22
	claudication		78 (19.7)	30 (22.7)	22 (16.7)	26 (19.8)	=	0.47
	tissue loss		8 (2)	3 (2.3)	1 (0.8)	4 (3.1)	=	0.41
	DVT		2 (0.5)	-	1 (0.8)	1 (0.8)	=	0.6
	ischemia	TASC I	44 (10.9)	18 (13.6)	14 (10.6)	11 (8.4)		0.56
		TASC IIa	26 (6.8)	9 (6.8)	10 (7.6)	9 (6.8)		
		TASC IIb	34 (8.6)	9 (6.8)	13 (9.8)	12 (9.2)		
		TASC IIII	2 (0.5)	-	-	2 (1.5)		

Table S1: Patient and PAA characteristics by consecutive thirds. Values presented as absolute numbers and percentage or mean ± one standard deviation; ASA= American society of anesthesiology, BMI= body mass index, CAD = coronary heart disease, renal insufficiency = serum creatinine >1.2 mg/dl COPD = chronic obstructive pulmonary disease; PAOD = peripheral arterial occlusive disease; AAA = Abdominal Aortic Aneurysm; TAA =

thoracic aortic aneurysm; ASS=aspirin; ACE=angiotensin converting enzyme; CRP=C reactive protein, Hb= hemoglobin; DVT = deep vein thrombosis; TASC = Transatlantic Society Consensus classificiation of acute limb ischemia; chi square or 1-way anova test to compare cohorts, $p<0.05$ is considered significant and highlighted bold; * calculation based on numbers given (*: 303 procedures: 82.8%)

			**combined**<br>n=29	elective<br>n=27	emergency<br>n=2
**patient characteristics**					
		age (years; mean ± SD)	71.6 ± 8.1	71.1 ± 7.8	78.5 ±13.4
		sex (male: N, %)	27 (93.1)	25 (92.3)	2 (100)
comorbidities		obesity (BMI > 30)	5 (17.2)	5 (18.5)	-
comorbidities		hypertension	22 (75.8)	21 (77.8)	1 (50)
comorbidities		diabetes	2 (6.8)	2 (7.4)	-
comorbidities		hyperlipidemia	17 (58.6)	17 (58.6)	-
comorbidities		CAD	16 (55.2)	15 (55.6)	1 (50)
comorbidities		renal insufficiency	3 (11.5)	3 (10.3)	-
comorbidities		dialysis	-	-	-
comorbidities		COPD	1 (3.4)	1 (3.7)	-
comorbidities		PAOD	8 (27.5)	8 (29.6)	-
comorbidities		malignancy	4 (13.8)	3 (11.1)	1 (50)
		nicotine abuse (current)	8 (27.6)	8 (29.6)	-
**PAA characteristics**					
		diameter (mm; mean ± SD)	31.7 ± 15.2	31.4 ± 15.7	35.5 ± 7.8
		tibial runoff vessels * (#)	1: 5 (20.8)<br>2: 6 (25)<br>3: 13 (54.2)	1: 5 (21.7)<br>2: 5 (21.7)<br>3: 13 (56.5)	1: -<br>2: 1 (100)<br>3: -
		symptomatic	9 (31)	7 (25.9)	2 (100)
symptoms		rupture	1 (3.4)	-	1 (50)
symptoms		local pain	5 (17.2)	4 (14.8)	1 (50)
symptoms		claudication	2 (6.9)	2 (7.4)	-
symptoms	ischemia	TASC I	1 (3.4)	1 (3.7)	-
symptoms	ischemia	TASC IIa	-	-	-
symptoms	ischemia	TASC IIb	1 (3.4)	-	1 (50)
symptoms	ischemia	TASC III	-	-	-
**procedural details**					
		procedure time	108.1 ± 92.6	99.3 ± 77.7	213 ± 227.7
		additional procedures	15 (51.7)	13 (48.1)	2 (100)
		conversion to OPAR	2 (6.8)	1 (3.7)	1 (50)

Table S2: Primary EPAR indication, patient, PAA characteristics and procedural details. Values presented as absolute numbers and percentage or mean ± one standard deviation; BMI= body mass index, CAD = coronary heart disease, renal insufficiency = serum creatinine >1.2 mg/dl, COPD = chronic obstructive pulmonary disease; PAOD = peripheral arterial occlusive disease; DVT = deep vein thrombosis; TASC = Transatlantic Society Consensus classificiation of acute limb ischemia; EPAR = endovascular PAA repair, OPAR = open PAA repair; * calculation based on numbers given (*: 24 procedures: 83.7%)

		**Combined**<br>n= 366	1st third<br>n= 122	2nd third<br>n= 122	3rd third<br>n= 122	trend	p
**procedural details**							
	procedure time (min, median)	246 [235-259]	225 [209-247]	263 [242-297]	259 [242-287]	↑	**0.002**
bypass configuration	medial access (vs. dorsal)	316 (86.3)	118 (96.7)	89 (73)	109 (89.3)	=	**<0.001**
bypass configuration	popliteo – popliteal	223 (60.9)	76 (62.2)	74 (60.7)	73 (59.8)		
bypass configuration	distal origin – popliteal	66 (18.0)	18 (14.8)	25 (20.5)	23 (18.9)	=	0.16
bypass configuration	crural bypass	65 (17.8)	23 (18.9)	19 (15.6)	23 (18.9)		
bypass configuration	*no successful bypass*	13 (3.6)	5 (4.1)	4 (3.3)	4 (3.3)	=	0.93
bypass configuration – material	saphenous vein	281 (76.8)	84 (68.9)	100 (82)	97 (79.5)		
bypass configuration – material	alloplastic	64 (18)	29 (23.8)	16 (13.1)	19 (15.6)	=	0.32
bypass configuration – material	composite	9 (2.5)	4 (3.4)	2 (1.7)	3 (2.5)		
add-on procedures	lysis pre-/intra-op	28 (7.8)	12 (9.8)	10 (8.2)	6 (4.9)	=	0.15
add-on procedures	embolectomy	76 (20.7)	20 (16.4)	27 (22.1)	29 (23.8)	↑	0.43
add-on procedures	local TEA	31 (8.4)	8 (6.5)	16 (13)	7 (5.7)	=	0.079
add-on procedures	PTA/stent (up-/downstream)	37 (10.1)	8 (6.5)	14 (11.4)	15 (12.3)	↑	0.27
add-on procedures	immediate revision	57 (15.5)	20 (16.4)	16 (13.1)	21 (17.2)	=	0.47
add-on procedures	additional (jump) graft	9 (2.4)	3 (2.4)	4 (3.3)	2 (1.6)	=	0.71
add-on procedures	fasciotomy	32 (8.7)	6 (4.9)	13 (10.6)	13 (10.7)	↑	0.12
**hemodynamic changes**							
	runoff vessels postoperative *	0: 15 (5)<br>1: 88 ()<br>2: 87 (28.7)<br>3:113 (37.3)	0: 5 (5.5)<br>1: 20 (22)<br>2: 25(27.5)<br>3:41 (45.1)	0: 4 (4.2)<br>1: 27 (28.4)<br>2: 23 (24.2)<br>3: 41 (43.2)	0: 6 (5.3)<br>1: 38 (33.3)<br>2: 39 (34.2)<br>3: 31 (27.2)	↓	0.059
	Δ runoff (# pre/post)	0.27 ± 0.96	0.42 ± 1.1	0.21 ± 0.92	0.21 ± 0.9	=	0.73
	Δ ABI (pre/post)	0.18 ± 0.35	0.19 ± 0.34	0.2 ± 0.38	0.15 ± 0.31	=	0.58
**postoperative course (in-hospital)**							
	hospital stay (d; median)	8 [8-10]	10 [10-12]	7 [7-9]	7 [7-9]	=	0.055
complications	surgical<br>bleeding<br>compartment syndrome<br>nerve lesion<br>SSI	104 (28.5)<br>12 (3.3)<br>8 (2.2)<br>5 (1.4)<br>63 (17.4)	40 (38.5)<br>3 (2.5)<br>4 (3.3)<br>1 (0.8)<br>24 (19.8)	33 (27)<br>6 (4.9)<br>-<br>4 (3.3)<br>21 (17.2)	31 (25.4)<br>3 (2.5)<br>4 (3.3)<br>-<br>18 (14.8)	↓	0.38
complications	medical<br>cardiovascular<br>pulmonary<br>acute kidney failure<br>other	42 (11.5)<br>15 (4.1)<br>8 (2.2)<br>10 (2.7)<br>24 (6.5)	12 (9.9)<br>5 (4.1)<br>-<br>2 (1.6)<br>9 (7.5)	12 (9.9)<br>3 (2.4)<br>3 (2.4)<br>4 (3.3)<br>5 (4.1)	18 (14.8)<br>7 (5.7)<br>5 (4.1)<br>4 (3.3)<br>10 (8.2)	↑	0.39
re-operation	hemorrhage	12 (3.3)	3 (2.5)	6 (4.9)	3 (2.5)	=	0.47
re-operation	compartment syndrome	7 (1.9)	4 (3.3)	-	3 (2.5)	=	0.15
re-operation	bypass occlusion	9 (2.2)	3 (2.5)	-	6 (4.9)	↑	**0.047**
re-operation	bypass stenosis	7 (1.9)	3 (2.5)	2 (1.6)	2 (1.6)	=	0.863
re-operation	aneurysm resection	3 (0.8)	2 (1.6)	-	1 (0.8)	=	0.36
re-operation	amputation (major)	14 (3.8)	2 (1.6)	4 (3.3)	8 (6.6)	↑	0.12
re-operation	wound revision	43 (11.8)	14 (11.5)	17 (13.8)	12 (9.8)	=	0.61
**post-discharge course (follow-up)**							
re-operation	hemorrhage	6 (1.6)	3 (2.5)	1 (0.8)	2 (1.7)	=	0.61
re-operation	bypass occlusion/stenosis	98 (26.8)	50 (41)	29 (23.8)	19 (15.6)	↓	**<0.001**
re-operation	aneurysm resection	17 (4.6)	8 (6.7)	5 (4.2)	4 (3.4)	=	0.47
re-operation	amputation	6 (1.7)	5 (4.2)	-	1 (0.9)	=	**0.031**
re-operation	wound revision	34 (9.2)	12 (10)	10 (8.3)	12 (10.4)	=	0.85

Table S3: Procedural details OPAR by consecutive thirds. Values presented as absolute numbers and percentage, mean ± one standard deviation or median with interquartile range [IQR]; three patients had conversion from dorsal to medial access (shown with medial); TEA = thrombendarterectomy, PTA = percutaneous transluminal

angioplasty, ABI=ankle brachial index; chi square or 1-way ANOVA test to compare cohorts, $p<0.05$ is considered significant and highlighted bold;. * calculation based on numbers given (*: 303 procedures: 82.8%);

		univariate				multivariate		
		**regression coefficient (Δmin)**	**95%-confidence interval**	**p**	x	**regression coefficient (Δmin)**	**95%-confidence interval**	**p**
**patient characteristics**								
age (+1 year)		+ 0.23	-0.86 – 1.33	0.69				
obesity		+ 17.6	-11.3 – 46.7	0.23				
comorbidities > 2		+ 12.1	-16.9 – 40.9	0.41				
any additional aneurysm		- 13.9	-36.6 – 8.7	0.23				
**PAA characteristics**								
diameter	(+2 mm)	+ 0.54	-1.4 – 2.5	0.58				
	(>30 mm)	+ 5.7	-17.3 – 28.7	0.63				
emergency		**+ 52.7**	**23.7 – 81.8**	**<0.001**	x	**42.6**	**9.9 – 75.3**	**0.01**
tibial runoff (0/1 vs 2/3 vessels)		**+ 59.5**	**34.6 – 84.4**	**<0.001**	x	**34.9**	**11.9 – 57.9**	**0.003**
symptomatic		**+ 30.1**	**7.2 – 53.1**	**0.01**	x	-1.2	-24.9 – 22.5	0.92
**procedural details**								
popliteo-popliteal (vs other)		**- 61.9**	**-84.1 - (-39.69)**	**<0.001**	x	**-45.9**	**-68.4 – (-23.5)**	**<0.01**
saphenous vein (vs other)		**+ 31.4**	**4 – 58.8**	**0.025**	x	**51.4**	**24.3 – 78.6**	**<0.01**
any additional procedure (+1)		**+ 78.5**	**57.1 – 99.8**	**<0.001**	x	**70.1**	**45 – 95.2**	**<0.01**
immediate revision		**+ 51.42**	**20.9 – 81.9**	**0.01**	x	3.6	-27 – 34.4	0.82

Table S4: Univariate and multivariate operating time analysis of all 366 OPAR procedures. x = Inclusion in the multivariate model; $p<0.05$ is considered significant and highlighted bold;

		Combined n=366	1. n=122	2. n=122	3. n=122	trend	p
**major amputation**							
	in-hospital	14 (3.8)	2 (1.6)	4 (3.3)	8 (6.6)	↑	0.124
	1 year	18 (4.9)	5 (4.1)	4 (3.3)	9 (7.4)	=	0.247
	5 years	18 (4.9)	5 (4.1)	4 (3.3)	9 (7.4)	=	
	overall	20 (5.5)	7 (5.8)	4 (3.3)	9 (7.4)	=	0.734
**mortality**							
	in-hospital	3 (0.8)	-	-	3 (2.5)	↑	0.048
	1 year	15 (4.1)	8 (6.6)	2 (1.6)	5 (4.1)	=	0.191
	5 years	47 (12.8)	24 (19.6)	13 (10.7)	10 (8.2)	↓	
	overall	82 (22.5)	49 (40.5)	20 (16.4)	13 (10.7)	↓	**<0.001**
**patency**							
primary	in- hospital	344 (97.5)	118 (97.5)	122 (100)	116 (95.1)	=	
	1 year	325 (92.1)	104 (85.2)	114 (93.4)	105 (86.1)	=	0.056
	5 years	297 (84.1)	91 (74.6)	104 (85.2)	102 (85.7)	=	
	overall	284 (80.2)	82 (70.3)	100 (84.7)	102 (85.7)	↑	**0.003**
sec	1 year	352 (96.2)	117 (95.9)	119 (97.5)	116 (95.1)	=	0.1
	5 years	348 (95.1)	115 (94.3)	119 (97.5)	114 (93.4)	=	
	overall	333 (93.3)	106 (89.8)	116 (96.7)	111 (93.3)	=	0.11

Table S5: OPAR outcome by consecutive cohorts. EPAR = endovascular PAA repair, OPAR = open PAA repair, sec = secondary; , p<0.05 is considered significant and highlighted bold;

		univariate				multivariate		
		**Odds ratio**	**95%-confidence interval**	**p**	x	**Odds ratio**	**95%-confidence interval**	**p**
**patient characteristics**								
age (+ 1 year)		**0.95**	**0.93 – 0.97**	**<0.01**	x	**0.96**	**0.93 – 0.98**	**<0.01**
comorbidities > 2		0.56	0.3 – 1.05	0.07				
somking (active)		1.37	0.8 – 2.36	0.25				
ASA (>III)		0.68	0.45 – 1.1	0.68				
any additional aneurysm		**0.63**	**0.4 – 0.99**	**0.04**	x	0.64	0.39 – 1.05	0.08
**PAA characteristics**								
diameter	(+2 mm)	0.99	0.96 – 1.04	0.89				
	(>30 mm)	1.3	0.83 – 2.1	0.24				
emergency		**0.42**	**0.224 – 0.72**	**0.02**	x	0.8	0.41 – 1.57	0.52
symptomatic		0.81	0.52 – 1.29	0.39				
**procedural details**								
operating time (+15 min)		1.02	0.99 – 1.06	0.16				
EPAR (vs OPAR)		0.84	0.35 – 1.98	0.69				
popliteo-popliteal (vs other)		1.23	0.75 – 2.03	0.4				
saphenous vein (vs other)		**1.87**	**1.16 – 3**	**0.01**	x	1.4	0.84 – 2.4	0.19
any additional procedure (+1)		0.92	0.73 – 1.17	0.49				
**postoperative course (in-hospital)**								
any complication		**0.43**	**0.27 – 0.69**	**<0.01**	x	0.83	0.38 – 1.81	0.64
complication: surgical		0.65	0.4 – 1.06	0.09				
complication: medical		**0.19**	**0.1 – 0.38**	**<0.01**	x	**0.29**	**0.12 – 0.7**	**<0.01**
any re-operation		**0.49**	**0.28 – 0.86**	**0.01**	x	0.68	0.29 – 1.58	0.37
bypass revision		0.49	0.15 – 1.58	0.23				
**post-discharge course (follow-up)**								
any re-operation		0.8	0.49 – 1.31	0.38				

Table S6: Univariate and multivariate outcome analysis on primary endpoint "amputation-free survival" of entire PAA cohort. ASA = American society of anesthesiology; EPAR = endovascular PAA repair, OPAR = open PAA repair; x = inclusion in the multivariate model; p<0.05 is considered significant and highlighted bold;
